# Comparison of fresh and frozen-thawed embryo transfer cycles in patients with low oocyte retrieval

**DOI:** 10.12669/pjms.40.10.9269

**Published:** 2024-11

**Authors:** Hongzhi Shi, Qi Song, Jiajia Liu, Chen Li, Rongrong Liu

**Affiliations:** 1 Hongzhi Shi, Department of Reproductive Medicine, Maternity & Child Care Center of Qinhuangdao, Qinhuangdao 066000, Hebei, China; 2 Qi Song, Department of Reproductive Medicine, Maternity & Child Care Center of Qinhuangdao, Qinhuangdao 066000, Hebei, China; 3 Jiajia Liu, Department of Reproductive Medicine, Maternity & Child Care Center of Qinhuangdao, Qinhuangdao 066000, Hebei, China; 4 Chen Li, Department of Reproductive Medicine, Maternity & Child Care Center of Qinhuangdao, Qinhuangdao 066000, Hebei, China; 5 Rongrong Liu, Children Protect Branch, Maternity & Child Care Center of Qinhuangdao, Qinhuangdao 066000, Hebei, China

**Keywords:** Low oocyte retrieval, Fresh embryo transfer cycle, Frozen embryo transfer cycle, Pregnancy outcome

## Abstract

**Objective:**

To explore the choice of embryo transfer schemes for patients with low oocyte retrieval (≤ 3 oocytes).

**Methods:**

A retrospective analysis was conducted on patients with oocyte yields ≤ 3 undergoing in vitro fertilization and embryo transfer (IVF-ET) and frozen embryo transfer (FET) at the Maternity & Child Care Center of Qinhuangdao Reproductive Medicine Department from January 2018 to December 2022. The data included 202 fresh cycles, with 104 cycles in Group-A and 98 cycles in Group-B. Additionally, 87 cycles involved the transfer of frozen embryos from fresh cycles that could not be transplanted for various reasons, with 31 cycles in Group-C and 56 cycles in Group-D. General patient information, embryo transfer details, and clinical pregnancy outcomes in both fresh and frozen cycles were statistically analyzed.

**Results:**

No significant differences were observed between Groups A and C in age, anti-Müllerian hormone (AMH), basal follicle-stimulating hormone (bFSH), body mass index (BMI), duration of infertility, the proportion of patients with diminished ovarian reserve (DOR), oocyte retrieval count, usable embryo count, or the number of transplanted embryos (all p> 0.05). Advanced age was a risk factor for a decreased pregnancy rate, and FET significantly increased the pregnancy rate (p< 0.05, respectively).

**Conclusion:**

For patients under 35 years old with low oocyte retrieval, it is recommended to freeze all embryos when available and then proceed with FET. For patients aged 35 and above, without reducing the pregnancy rate, fresh embryo transfer is recommended to minimize treatment cycle frequency and economic expenses.

## INTRODUCTION

In assisted reproductive technology (ART), the number of retrieved oocytes after ovulation induction treatment is an important factor influencing patients’ pregnancy outcomes. Generally, it is considered appropriate to retrieve 6–15 oocytes, with optimal pregnancy outcomes observed in patients retrieving 9–12 oocytes.^1^–^3^ Poor ovarian response (POR) may result in a low number of retrieved oocytes (≤ 3 oocytes), and possible causes include advanced age, obesity, ovarian surgery, or ovarian dysfunction due to cancer radiotherapy and chemotherapy.^4^-^7^ A low number of retrieved oocytes can lead to a decrease in fertilized oocytes and usable embryos, an increase in cycle cancellation rates, a decline in cumulative pregnancy rates per retrieval cycle, and consequently, an increase in the physical, mental, and financial burden on patients.^8^ While previous literature has mainly compared the outcomes between low and normal oocyte retrieval numbers^1^, there is limited research specifically comparing patients with low oocyte retrieval counts. By investigating the differences between fresh embryo transfer and frozen embryo transfer (FET) in patients with low oocyte retrieval counts when usable embryos are available, this study aimed to determine a more suitable transfer scheme for patients with low oocyte retrieval.

## METHODS

A retrospective analysis was conducted on patients undergoing in vitro fertilization and embryo transfer (IVF-ET) treatment at the Reproductive Medicine Department of Maternity & Child Care Center of Qinhuangdao from January 2018 to December 2022. The data included 202 fresh cycles, with 104 cycles in Group-A and 98 cycles in Group-B. Additionally, 87 cycles involved the transfer of frozen embryos from fresh cycles that could not be transplanted for various reasons, with 31 cycles in Group-C and 56 cycles in Group-D. General patient information, embryo transfer details, and clinical pregnancy outcomes in both fresh and frozen cycles were statistically analyzed. The study focused on patients with an oocyte retrieval count of ≤3.

### Ethical Approval:

The study was approved by the Institutional Ethics Committee of Maternity & Child Care Center of Qinhuangdao (No.: QHDFY-2023031008; date: March 11, 2023), and written informed consent was obtained from all participants.

### Inclusion criteria:

- Oocyte retrieval count ≤ 3 in fresh retrieval cycles;
- Aged 26~45 years old.
- Availability of usable embryos on day three (D3) for transfer or complete embryo cryopreservation
- Frozen embryo transfer (FET) cycles involving the first transfer after complete embryo cryopreservation.

### Exclusion criteria:

- Uterine malformations or endometriosis during the transfer cycle;
- Blastocyst transfers.

### Controlled ovarian stimulation and embryo culture

Individualized ovarian stimulation protocols were developed based on patient age, ovarian reserve, and other relevant factors. Oocytes were retrieved under transvaginal ultrasound guidance 36 hours after human chorionic gonadotropin (HCG) injection. Male partners were provided semen samples on the day of oocyte retrieval through masturbation. After liquefaction, semen was processed using the density gradient centrifugation method with Pureception (CooperSurgical, US). Washing and culture media for sperm, fertilization, cleavage, and blastocyst development were all provided by the Sage medium (CooperSurgical, US). Fertilization was performed in a dual-chamber dish 39–40 hours post-HCG injection. After 17–19 hours, pre-implantation genetic assessment was conducted. Fertilized embryos were cultured until D3, and following 66–70 hours post-fertilization, embryos were assessed according to the Association for the Study of Reproductive Biology (ASEBIR) cleavage-stage embryo grading system^9^. Embryos were classified into grades I–IV, with grades I–III indicating normally cleaved embryos for selective transfer or cryopreservation. Cryopreservation and thawing of embryos were performed using freezing and thawing kits (Kitazato, Japan). All operations were performed by the same group of doctors.

### Fresh embryo transfer:

On D3, embryos classified as grade III or IV were selected for transfer. Using a peripherally inserted central catheter, embryos were transplanted into the posterior part of the uterine cavity under ultrasound guidance. Clinical pregnancy was confirmed by the presence of a gestational sac and fetal heart on abdominal ultrasound 35 days post-transfer.

### Frozen embryo transfer (FET):

To avoid confounding factors due to embryo quality, all FETs involved the first cycles for patients with complete embryo cryopreservation. According to each patient’s individual circumstances, 1–2 thawed embryos were transplanted after endometrial transformation. Patients were divided into four groups by age and type of transfer cycle: (1) Group A: < 35 years old, fresh embryo transfer; (2) Group B: ≥ 35 years old, fresh embryo transfer; (3) Group C: < 35 years old, FET; (4) Group D: ≥ 35 years old, FET.

### Observation indicators and criteria:

- Clinical pregnancy rate = Number of cycles with detectable gestational sacs under ultrasound / Number of transfer cycles × 100%.
- Implantation rate = Number of detected gestational sacs under ultrasound / Number of transplanted embryos × 100%.
- Miscarriage rate = Number of miscarriage cycles / Number of clinical pregnancy cycles × 100%.

### Statistical analysis:

SPSS 23.0 was used for statistical analysis. For normally distributed measurement data, one-way analysis of variance was conducted, and results were presented as “mean ± standard deviation (*x̅*±*s*)”. The confidence interval was 95%, non-normally distributed measurement data were compared using the rank-sum test, with results expressed as median (25%, 75%). Pairwise comparisons were made using t-tests. Enumeration data were presented as percentages (%) and compared between groups using the chi-square test or Fisher’s exact test. Binary logistic regression analysis was employed to calculate odds ratios (OR), with the level of statistical significance set at p< 0.05.

## RESULTS

Group-A consisted of 104 fresh embryo transfer cycles for patients under 35 years old, while Group-C comprised 31 FET cycles for patients under 35 years old. The two groups showed no significant differences in baseline characteristics, including age, anti-Müllerian hormone (AMH), basal follicle-stimulating hormone (bFSH), body mass index (BMI), duration of infertility, the proportion of patients with diminished ovarian reserve (DOR), and laboratory indicators such as the number of retrieved oocytes, usable embryos, and transplanted embryos (all p> 0.05). However, compared to Group-A, Group-C exhibited a significantly higher proportion of primary infertility and clinical pregnancy outcomes, including clinical pregnancy rate and implantation rate (p< 0.05, respectively). No significant difference was observed in early miscarriage rates between the two groups (p> 0.05) (Table-I).

**Table-I:** Comparison of baseline characteristics and clinical pregnancy outcomes after embryo transfer between Groups A and C [(M (P25, P75), (*x̅*±*s*), %)].

Group		Number of Cycles	Age	AMH		bFSH		BMI	Duration of Infertility		Type of Infertility				Patients with DOR (%)

											Primary (%)		Secondary (%)		
Group A		104	30.64±2.41	0.91 (0.48, 1.52)		9.90±4.98		23.84±4.51	3.87±2.55		52.88 (55/104)		47.12 (49/104)		52.88 (55/104)
Group C		31	31.06±2.89	0.86 (0.45, 1.93)		9.29±4.48		24.02±5.44	3.87±2.70		74.19 (23/31)*		25.00 (9/36)*		51.61 (16/31)
*t/X^2^*			0.663	0.025		0.058		0.030	0.000		6.887		6.887		0.111
*P*			0.417	0.875		0.810		0.862	0.998		0.009		0.009		0.840

***Group***	***Number of Retrieved Oocytes***				***Usable Embryos***		***Transplanted Embryos***			***Pregnancy Rate***		***Implantation Rate***		***Early Miscarriage Rate***	

Group A	2.34±0.71				1.63±0.67		1.49±0.52			32.69 (34/104)		24.52 (38/155)		5.88 (2/34)	
Group C	2.23±0.80				1.39±0.56		1.42±0.50			54.84 (17/31)*		40.91 (18/44)*		11.76 (2/17)	
*X^2^*	0.551				3.226		0.451			4.865		4.554		0.514	
*P*	0.459				0.075		0.503			0.035		0.033		0.593	

***Note:***

* p< 0.05 compared to Group A.

Group-B included 98 fresh embryo transfer cycles for patients aged 35 and above, while Group-D comprised 56 FET cycles for patients aged 35 and above. Comparisons of baseline characteristics, including age, AMH, bFSH, BMI, duration of infertility, the proportion of primary infertility, and the proportion of patients with DOR, revealed no significant differences between the two groups (all p> 0.05). Laboratory indicators, including the number of retrieved oocytes, usable embryos, transplanted embryos, and clinical pregnancy outcomes (pregnancy rate, implantation rate, and early miscarriage rate), also showed no significant differences between Groups-B and D (all p> 0.05) (Table-II).

**Table-II:** Comparison of baseline characteristics and clinical pregnancy outcomes after embryo transfer between Groups B and D [(*χ̅*±*s*), %]

Group	Number of Cycles	Age		AMH		FSH	BMI		Duration of Infertility	Type of Infertility		Patients with DOR (%)

										Primary (%)	Secondary (%)	
Group B	98	39.38±3.44		1.12±1.38		9.83±4.33	24.30±3.82		4.53±4.10	36.73 (36/98)	63.27 (62/98)	66.33 (65/98)
Group D	56	40.07±4.33		0.90±1.46		9.82±4.16	24.54±3.44		3.86±3.11	39.29 (22/56)	60.71 (34/56)	75.00 (42/56)
*t/X^2^*		1.199		0.873		0.000	0.145		1.119	0.099	0.099	1.264
*P*		0.275		0.352		0.985	0.704		0.292	0.753	0.753	0.261

***Group***	***Number of Retrieved Oocytes***		***Usable Embryos***		***Transplanted Embryos***			***Pregnancy Rate (%)***		***Implantation Rate***		***Early Miscarriage Rate***

Group B	2.18±0.76		1.55±0.54		1.48±0.50			16.32 (16/98)		11.03 (16/145)		18.75 (3/16)
Group D	2.05±0.82		1.39±0.56		1.46±0.50			21.43 (12/56)		20.00 (14/82)		16.67 (2/12)
*t/X^2^*	0.980		2.971		0.033			0.642		1.665		1.000
*P*	0.324		0.087		0.856			0.430		0.197		0.583

To minimize confounding factors, binary logistic regression analysis was conducted, incorporating variables such as embryo transfer scheme, age, AMH, FSH, BMI, the number of DOR cases, and the number of retrieved oocytes. The results revealed that patient age and the choice of embryo transfer scheme were significant influencing factors for increasing clinical pregnancy rate. Compared to fresh embryo transfer, FET significantly increased the pregnancy rate [OR = 0.470, 95% CI (0.256–0.863), p< 0.05]. Additionally, the pregnancy rate decreased with the advancement of age [OR = 0.885, 95% CI (0.834–0.939), p< 0.05] (Table-III).

**Table-III:** Binary logistic regression analysis of factors influencing clinical pregnancy rate

Item	β	SE	Wald	P	OR	95%CI
Embryo transfer scheme	-0.755	0.310	5.939	0.015*	0.470	0.256–0.863
Age	-0.122	0.030	16.376	0.000*	0.885	0.834–0.939
AMH	0.035	0.085	0.167	0.683	1.036	0.876–1.224
FSH	0.015	0.032	0.240	0.624	1.016	0.955–1.080
BMI	0.045	0.033	1.838	0.175	1.046	0.980–1.117
Number of DOR cases	-0.306	0.300	1.041	0.308	0.737	0.409–1.325
Number of Retrieved Oocytes	0.292	0.195	2.249	0.134	1.339	0.914–0.961

***Note:***

* p< 0.05.

## DISCUSSION

In this study, the clinical pregnancy rate in fresh transfer cycles for patients with an oocyte retrieval count of ≤ 3 was 20.41%, significantly lower than those with oocyte retrieval counts of 5–8, 9–12, and 13–16. Notably, the highest pregnancy rate was observed in the patient group with 9–12 retrieved oocytes (52.11%) 1. Reproductive experts have reached a consensus — in an embryo transfer cycle, a lower oocyte retrieval count inevitably leads to a decrease in the number of usable embryos on D3, resulting in a reduction in the pregnancy rate.^10^

Studies^11^,^12^ have confirmed that FET cycles can yield a significantly higher pregnancy rate compared to fresh embryo transfer cycles. This might be explained by the substantial elevation of estrogen and progesterone levels during the ovarian stimulation process of a fresh embryo transfer cycle, which, as studies indicate, can alter the distribution and expression of cytokines, proteins, and transcription factors associated with embryo implantation, consequently affecting endometrial receptivity and hindering normal embryo implantation.^13^–^16^ In contrast, FET cycles, whether natural or artificial, have a minimal impact on estrogen and progesterone levels, improving endometrial receptivity and favoring embryo implantation.^17^ Limited usable embryos are particularly precious for patients with a low oocyte retrieval count. In light of this, the current study examined the utilization of these embryos, revealing that for patients under 35 years old, the pregnancy rate and embryo implantation rate in FET cycles were significantly higher than in fresh embryo transfer cycles. However, in patients aged 35 and above, no significant differences were observed in pregnancy and implantation rates between FET and fresh embryo transfer cycles. Through binary logistic regression analysis, patient age and the choice of embryo transfer scheme were identified as crucial factors affecting the pregnancy rate. Nevertheless, Feng LZ et al.^18^ argued that FET cycles did not improve pregnancy outcomes for patients with POR. This paradoxical conclusion may be attributed to the fact that Feng et al. did not group patients with an oocyte retrieval count of ≤ 3, and the average age of patients in the FET and fresh embryo transfer cycles (38.22 vs. 38.53) was similar to the older group in the present study. Therefore, their conclusion is consistent with our study results: FET and fresh embryo transfer cycles did not differ greatly in pregnancy rates.

The present study uncovers that, for patients under 35 years old, FET can improve pregnancy outcomes. However, when a patient reaches 35 or above, age becomes the predominant factor influencing pregnancy outcomes. A previous study^19^ reported age as an independent risk factor for patient pregnancy outcomes. With increasing age, ovarian reserve function declines, manifesting as a reduction in basal follicles and decreased ovarian response to ovulation-inducing drugs. Advanced age also alters the microenvironment of oocytes, thereby exerting an impact on the maturation and quality of oocytes. This leads to a decrease in fertilization and 2PN cleavage rates, compromised embryo development potential, an increased non-disjunction rate, and a reduced number of usable embryos. Additionally, in older patients, the decline in pregnancy rates is associated with a decrease in the number of estrogen receptors in the endometrium, reduced endometrial blood supply, and diminished endometrial receptivity.^20^–^21^ Therefore, changes in transfer techniques cannot improve pregnancy outcomes in older patients.

### Limitations

However, this study also has some shortcomings, such as a modest number of patients under 35 years old in the FET cycle group, potentially causing biased conclusions. Therefore, validation of the findings awaits further data from a larger sample size in subsequent studies.

## CONCLUSIONS

For patients under 35 years old with a low oocyte retrieval count, embryo cryopreservation and FET are recommended when usable embryos are available. However, for patients aged 35 and above, it is advisable to undergo fresh embryo transfer to reduce the number of treatment cycles and related expenses without compromising the pregnancy rate.

### Authors’ Contributions:

**HS and QS** carried out the studies, collected data, drafted the manuscript,

Accountable for the accuracy or integrity of the work.

**JL CL & RL:** performed the statistical analysis, and participated in its design.

All authors read and approved the final manuscript.
